# The Role of Research, Quality Improvement, and Training in Achieving Medicines Security in Nigeria: A Cross‐Sectional Exploration of Stakeholders' Perspectives

**DOI:** 10.1002/hsr2.72624

**Published:** 2026-08-04

**Authors:** Obi Peter Adigwe, Godspower Onavbavba, Toluwalope Folashade Alabi, Nwakaego Rosemary Uzuh

**Affiliations:** ^1^ National Institute for Pharmaceutical Research and Development Abuja Federal Capital Territory Nigeria

**Keywords:** development, healthcare, manufacturing, pharmaceuticals, production

## Abstract

**Background and Aims:**

Equitable access to medicines remains a challenge in Nigeria with issues such as sub‐optimal national pharmaceutical production capacities and the need to improve relevant quality standards. Thus, in order to identify context‐specific solutions to this challenge, this study aimed to assess the perspectives of stakeholders in the healthcare value chain on the role of quality improvement and capacity‐building strategies relevant to expediting medicines security in Nigeria.

**Methods:**

A national cross‐sectional survey was conducted in Nigeria using a multistage sampling technique. A pretested questionnaire was administered to relevant stakeholders in the health sector. Descriptive and inferential statistical analyses were undertaken using Statistical Package for Social Sciences (SPSS) software version 25.

**Results:**

A total of 416 questionnaires were retrieved. Just over a third of the study participants (37.9%) surveyed were actively employed in the pharmaceutical manufacturing sector. Nearly all the participants (94.8%) believed regulatory authorities should ensure the stringent implementation of Good Manufacturing Practices to sustain consistency in the quality of medicines. Similarly, over three‐quarters of the study cohort (86.1%) indicated that the pharmaceutical sector, as well as relevant agencies in the industry, should be prioritised by the government, as this was critical in achieving medicines security. Also, a strong majority of the participants indicated that attaining the World Health Organization pre‐qualification status was necessary to ensure access to high‐quality medicines. There was a significant association between the participants' sociodemographic characteristics and key study variables. A higher proportion of the respondents with more than 5 years of experience agreed that investment in research and development was important to improve the quality of medicines, and this was statistically significant (*p* = 0.009).

**Conclusion:**

This cross‐sectional study highlights strategic areas based on stakeholders' perceptions where interventions and reforms can be channelled to support efforts towards expediting the achievement of medicines security in Nigeria.

## 1 Introduction

Medicines security is an emergent concept that advocates for proactive control of pharmaceutical production and supply chain systems as a means of improving sustainable access to high‐quality, affordable healthcare for the population [1]. The concept is similar to access to medicines, which supports supply chain resilience and addresses the health system's ability to withstand disruptions [2]. Furthermore, it aligns with national self‐reliance and control over domestic manufacturing systems [3], as it focuses on developing capacity for reliable access and emphasises long‐term sustainability. In essence, medicines security is conceptualised as an outcome influenced by supportive policies, regulations, expansion of local manufacturing capacity, research and development for quality improvement, and workforce strengthening [4, 5].

Challenges in achieving medicines security persist despite global efforts to ensure “access to safe, effective, high‐quality, and affordable pharmaceutical products for all” as highlighted by the United Nations Sustainable Development Goal 3 [6]. Globally, approximately a third of the population lacks access to essential medicines, with regional disparities observed across continents [7]. In Africa, the situation is exacerbated by inadequate funding, the prevalence of counterfeit medicines, and supply chains that are highly reliant on imports [8]. Historically, the scenario is not different in Nigeria, where there are concerns of national pharmaceutical production capacity falling short of international quality standards as well as not meeting the healthcare supply needs of citizens [9, 10, 11]. These limitations have, over the years, contributed to inequitable access to medicines in the country, which ultimately increases public health risks given the high population and disease burden [12]. Recent empirical hospital‐level evidence further indicates that regulatory challenges and manufacturing delays constitute one of the largest contributors to a lack of access to medicines. This accounts for 39% of cases, thereby placing additional strain on health systems and continuity of patient care [13]. These shortfalls highlight the crucial need for contextual capacity improvement in the pharmaceutical manufacturing sector.

A search of the extant literature revealed that several factors have been associated with the availability and affordability of medicines. Available evidence highlights the significance of strengthening regulatory frameworks within the pharmaceutical sector and ensuring enforcement mechanisms for compliance with regulatory standards [14]. Extant literature also identifies a strong need for global health diplomacy through universal and regional collaborations, especially between public and private sectors [7, 15, 16].

At the 74th United Nations General Assembly, it was recommended that tackling the challenges of equitable access to medicines would involve reducing medicine costs by lowering taxes, abolishing tariffs, and eradicating other trade barriers [17]. Furthermore, the World Health Organization (WHO) emphasised the need for governments to assume greater responsibility for health sector interventions; reduce reliance on donor agencies; and address infrastructural needs [18]. Tackling medicines security issues in Nigeria will require a multifaceted approach that addresses the challenges militating against access [19]. However, no study has comprehensively assessed the perspectives of stakeholders in this regard despite its criticality. It is against this backdrop that this study was conceptualised. Therefore, in order to identify context‐specific solutions to the problems of equitable access to medicines in Nigeria, this study aimed to assess the perspectives of relevant stakeholders in the healthcare value chain.

## 2 Methods

This national study adopted a cross‐sectional design, which was undertaken between September and December 2024 amongst stakeholders in the Nigerian health sector. The data collection tool employed was a questionnaire developed in English language by a team of researchers following a comprehensive literature review [10, 18, 20]. Independently, each member of the team reviewed the items in the draft tool and suggested necessary changes. The revision process continued until a consensus was reached. The items in the questionnaire included sociodemographic characteristics and sections on the role of quality improvement and capacity‐building initiatives in expediting medicines security.

The research instrument validation was carried out by a team of five experts, specifically selected based on their professional experience in pharmaceutical regulation, manufacturing, health policy, and research, each with over 10 years of sectoral experience. The experts restructured and reworded some of the questionnaire items during face validation. The questionnaire was assessed for appropriateness, complexity, attractiveness, and applicability in the subject area. Content validity was also tested for the research tool. This was evaluated by assessing the content validity index (CVI) and content validity ratio (CVR) of the questionnaire item using quantitative methods. Minimum threshold CVI of 0.80 and CVR of 0.99 were adopted based on established recommendations [21, 22]. In total, 19 statements passed the tests and were selected for inclusion into the final data collection instrument. Furthermore, a pilot study was undertaken with the administration of the questionnaire to 25 participants. This was to assess clarity, relevance, and feasibility of the instrument, whilst internal consistency was evaluated using Cronbach's alpha (*α* = 0.97). The feedback received from the pretesting process did not necessitate any major change.

A population size of 940,193, which represents the workforce in the health sector, was used to determine the sample size [23]. This was calculated using Epi Info software version 7, set at 95% confidence interval, 5% margin of error, and 50% response distribution, resulting in a sample size of 385. A stratified multistage sampling strategy was used to ensure a diverse and representative sample. In the first stage, Nigeria was stratified into its six geopolitical zones. In the second stage, the geopolitical zones were stratified into states. One state was randomly selected from each geopolitical zone to ensure national coverage. In the third stage, relevant health‐sector institutions and organisations within the selected states were identified. In the fourth stage, eligible respondents were purposively selected within these institutions based on predefined stakeholder categories, including healthcare professionals, pharmaceutical manufacturers, experts in the regulatory sector, researchers, and individuals in academia. Others were professionals involved in medicine supply chain management, procurement and logistics services, health financing, and insurance schemes. Within each category, participants were approached using a random sampling approach based on availability and willingness to participate until the required sample size was achieved. The inclusion criteria for participating in this study were Nigerians practising in the aforementioned settings, who were 18 years or above and were willing to provide informed consent as well as participate in the study. Respondents who did not meet these criteria were excluded from the study.

Before the data collection process, ethical approval was sought and obtained from the National Institute for Pharmaceutical Research and Development Health Research Ethics Committee (Approval Number: HREC 09/11/2024‐33). Each participant provided a written informed consent that validated their voluntary participation. To ensure confidentiality, the questionnaire was designed without any fields for names or other identifying information, and no such data were collected. Participation was entirely voluntary, with respondents completing the survey independently. Access to completed questionnaires and raw data was restricted to the research team. All analyses and reporting were performed using aggregated data, with no disclosure of information that could directly or indirectly identify individual participants or institutions.

To further minimise response and social desirability bias, questions were framed neutrally without regulatory judgment, implications of blame, or attribution to specific institutions, thereby reducing any perceived pressure to provide socially desirable answers. Following the conclusion of data collection, the retrieved responses were coded into Statistical Package for Social Sciences (SPSS) version 25. Univariate analysis was undertaken to yield descriptive statistics, whilst Pearson's *χ* ^2^ test of independence was employed to determine the association between sociodemographic characteristics and the variables. Effect sizes for the *χ* ^2^ associations were estimated using Cramér's *V*. All statistical tests were two‐sided, and a *p* value of 0.05 or less represented the threshold for statistical significance. Prior to the analysis, assumptions for the *χ* ^2^ test were assessed, including independence of observations and adequacy of expected cell frequencies, and were found to be satisfied.

Incomplete responses were handled using available‐case analysis. Questionnaires with substantial missing data in key variables were excluded. The extent of missing data was minimal and unlikely to meaningfully influence the results.

## 3 Results

### 3.1 Demography

A total of 416 valid responses were retrieved following the data collection. Male participants (62.5%) were more than the female respondents (37.5%), and slightly above a third (38.2%) were employed in the pharmaceutical manufacturing sector. Further details about the sociodemographic characteristics of the participants are presented in Table 1.

**Table 1:** Sociodemographic characteristics of participants.

Demography	Frequency (%)
Gender	
Male	260 (62.5)
Female	156 (37.5)
Highest educational qualification	
Diploma	8 (1.9)
First degree	281 (67.5)
Master's degree	92 (22.1)
Doctorate	35 (8.4)
Age (years)	
18–30	186 (44.7)
31–40	87 (20.9)
41–50	59 (14.2)
51–60	68 (16.3)
Above 60	16 (3.8)
Area of employment	
Pharmaceutical manufacturing	159 (38.2)
Nonpharmaceutical manufacturing	257 (61.8)
Sector of practice	
Government sector	161 (38.7)
Private sector	223 (53.6)
Development agency	27 (6.5)
Others	5 (1.2)
Years of experience in your current work	
< 5	188 (45.2)
5–10	76 (18.3)
11–15	38 (9.1)
> 15	114 (27.4)

### 3.2 Strategies for Improving the Quality of Pharmaceutical Manufacturing in Nigeria

#### 3.2.1 Regulation of Pharmaceutical Manufacturing

**Figure 1:**
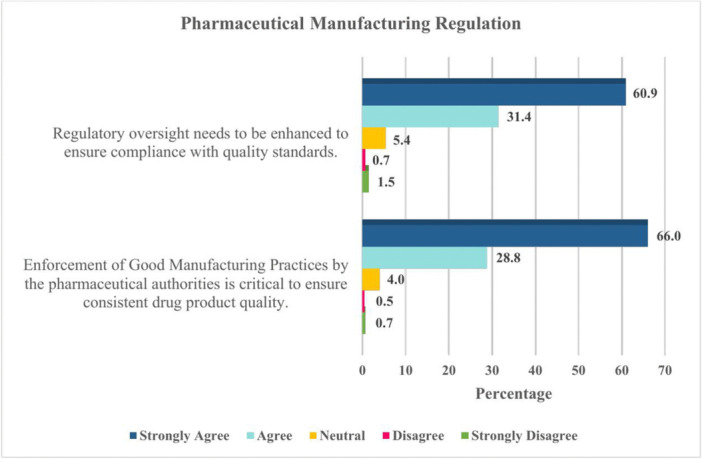
Pharmaceutical manufacturing regulation.

Findings presented in Figure 1 show that a strong majority of the respondents (92.3%) were of the view that enhancing regulatory compliance was necessary to uphold adherence to quality standards.

Similarly, almost all the participants (94.8%) held the view that regulatory authorities need to ensure stringent implementation of Good Manufacturing Practices (GMP) to sustain consistency in the quality of medicinal products.

#### 3.2.2 Favorable Economic Policies

Participants' opinions concerning favorable economic policies include catalysing standard pharmaceutical manufacturing through tax incentives, accessible foreign exchange, and local content prioritisation. In terms of tax incentives, more than three‐quarters of the respondents (88.8%) were of the belief that introducing relevant tax waivers for the purchase of equipment and raw materials can improve capacity towards local manufacturing of pharmaceutical products.

**Figure 2:**
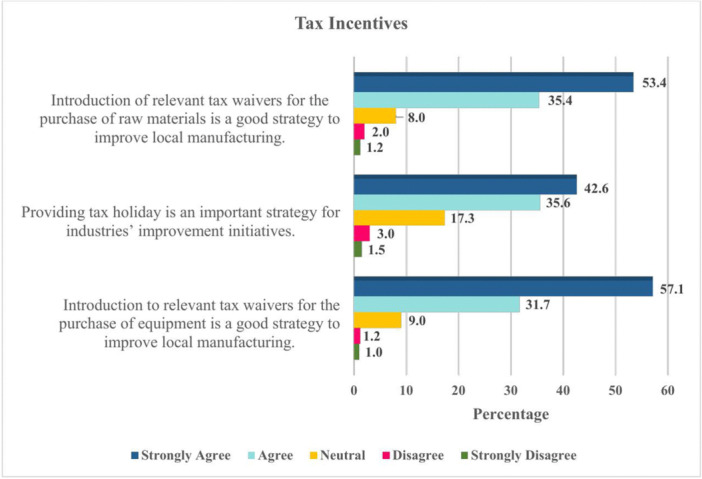
Tax incentives.

As presented in Figure 2, over three‐quarters of the participants (78.2%) indicated that considering incentives such as tax holidays represented a useful tool in promoting the growth and development of the pharmaceutical industry.

**Figure 3:**
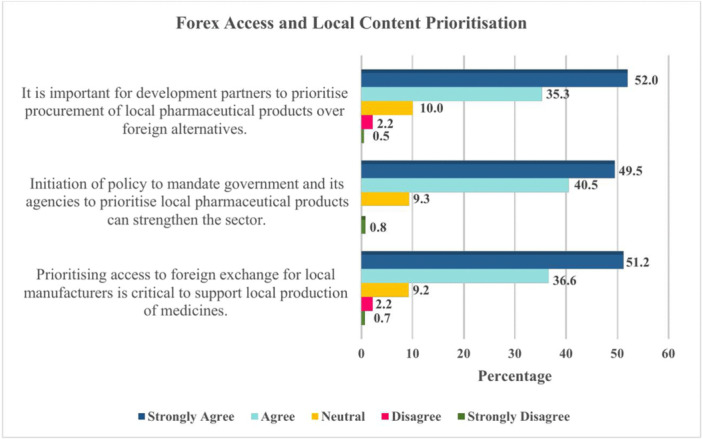
Forex and local content prioritisation.

Findings showed that a significant proportion of the sample (87.8%) believed that facilitating access to foreign exchange for local manufacturers was vital to support local manufacturing of medicines. Only a few of the participants (3.9%) indicated otherwise. Further details are presented in Figure 3.

A strong majority of the respondents (90.0%) indicated that policies should be established to encourage government and its agencies to make locally manufactured pharmaceutical products a priority. Additionally, more than three‐quarters of the participants (87.3%) indicated that it was important for development partners to give precedence to procuring local pharmaceutical products rather than foreign alternatives.

#### 3.2.3 Funding and Collaborations

**Figure 4:**
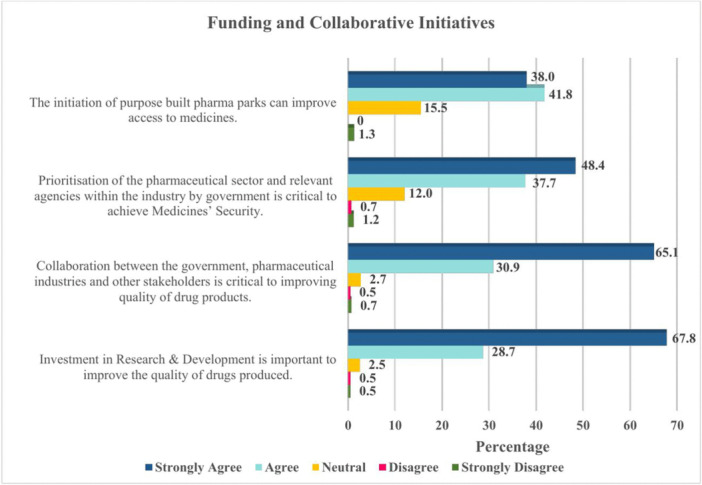
Funding and collaborative initiatives.

In the area of funding and collaborations, most of the participants (96.5%) were of the opinion that investments in research and development activities can help improve the quality standards of medicines. A similar proportion of the study cohort (96.0%) agreed that government, pharmaceutical industries, and other relevant stakeholders need to collaborate to advance the quality standards of medicines. Further details are presented in Figure 4.

Additionally, findings in Figure 4 show that over three‐quarters of the study participants (86.1%) indicated that the pharmaceutical sector and relevant agencies in the industry should be prioritised by the government, as this was critical in achieving medicines security.

### 3.3 Capacity Improvement Programmes

#### 3.3.1 Quality Assurance in Medicines

**Figure 5:**
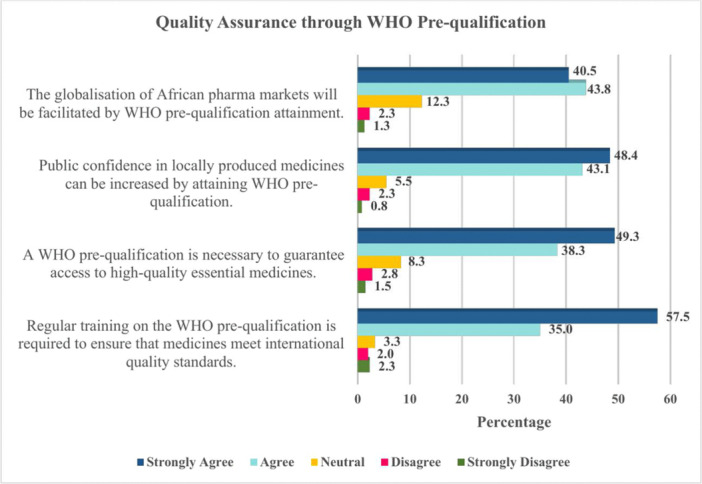
Quality assurance through WHO pre‐qualification.

In the aspect of quality assurance, almost all the participants (91.5%) indicated that attaining the WHO pre‐qualification status can increase public confidence in locally produced medicines. Also, a strong majority of the respondents (87.6%) were of the opinion that attaining this status was necessary to ensure access to high‐quality medicines. Further details are presented in Figure 5.

Furthermore, from Figure 5, almost all the participants (92.5%) indicated that regular training on processes relating to WHO pre‐qualification was critical for ensuring that medicines meet international quality standards.

#### 3.3.2 Capacity Building

**Figure 6:**
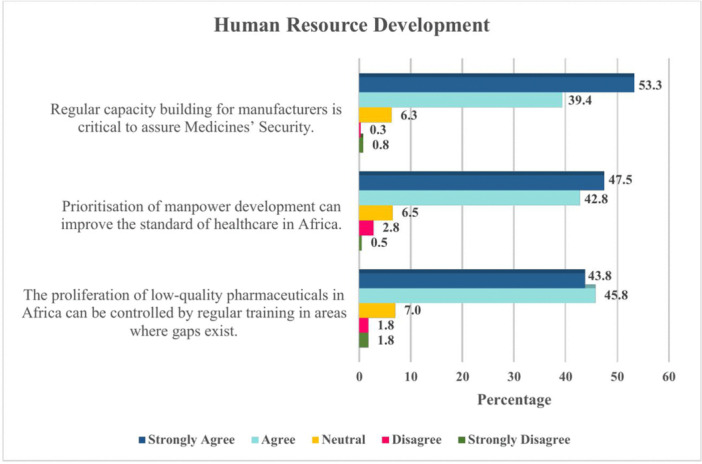
Human resource development.

Regarding capacity building, almost all of the participants (92.7%) felt that regular capacity building for manufacturers was critical to assure medicines security. Similarly, a strong majority (90.3%) of the respondents indicated that healthcare standards in Africa can improve if manpower development is prioritised. Other relevant details are presented in Figure 6.

Findings in Figure 6 show that the majority of the participants (89.6%) were of the view that the proliferation of low‐quality pharmaceuticals in Africa can be controlled by regular training in areas where gaps exist.

### 3.4 Association Between Variable and Sociodemographic Characteristics

*χ* ^2^ analysis was undertaken to determine the association between sociodemographic characteristics and participants' views that enforcement of GMP by relevant authorities would ensure consistent quality of pharmaceutical products. Participants' gender (*p* = 0.005, Cramér's *V* = 0.191), highest educational qualification (*p* < 0.001, *V* = 0.176), sector of practice (*p* < 0.001, *V* = 0.183), and years of practice (*p* = 0.035, *V* = 0.136) had significant associations with the statement. Effect sizes were calculated using Cramér's *V*, and the coefficients indicated low magnitude with values ranging from 0.136 to 0.183 for the significant variables. The proportion of participants who agreed with the statement progressively increased with higher educational qualifications. Other relevant details are presented in Table 2.

**Table 2:** Cross‐tabulation of sociodemographic characteristics with the statement “Enforcement of good manufacturing practices by pharmaceutical regulatory authorities is critical to ensure consistent drug product quality.”

Demography	Strongly Disagree (%)	Disagree (%)	Neutral (%)	Agree (%)	Strongly Agree (%)	*χ* ^2^	*p*	Cramer's *V*
Gender						14.665	0.005	0.191
Male	3 (1.2)	2 (0.8)	4 (1.6)	80 (31.3)	167 (65.2)			
Female	0 (0.0)	0 (0.0)	12 (8.2)	36 (24.5)	99 (67.3)			
Highest educational qualification						37.382	< 0.001	0.176
Diploma	1 (12.5)	0 (0.0)	0 (0.0)	6 (75.0)	1 (12.5)			
First degree	2 (0.7)	2 (0.7)	15 (5.5)	64 (23.5)	189 (69.5)			
Master's degree	0 (0.0)	0 (0.0)	1 (1.1)	32 (36.4)	55 (62.5)			
Doctorate	0 (0.0)	0 (0.0)	0 (0.0)	13 (38.2)	21 (61.8)			
Age (years)						22.782	0.120	0.119
18–30	2 (1.1)	2 (1.1)	15 (8.2)	48 (26.2)	116 (63.4)			
31–40	0 (0.0)	0 (0)	0 (0)	27 (31.8)	58 (68.2)			
41–50	1 (1.9)	0 (0)	1 (1.9)	14 (25.9)	38 (70.4)			
51–60	0 (0.0)	0 (0.0)	0 (0)	23 (35.4)	42 (64.6)			
Above 60	0 (0.0)	0 (0)	0 (0)	4 (25.0)	12 (75.0)			
Area of employment						4.277	0.370	0.103
Pharmaceutical manufacturing	0 (0.0)	0 (0.0)	5 (3.3)	49 (32.0)	99 (64.7)			
Nonpharmaceutical manufacturing	3 (1.2)	2 (0.8)	11 (4.4)	67 (27.0)	165 (66.5)			
Sector of practice						40.416	< 0.001	0.183
Government sector	1 (0.6)	0 (0.0)	8 (5.0)	52 (32.7)	98 (61.6)			
Private sector	2 (0.9)	0 (0.0)	5 (2.3)	55 (25.8)	151 (70.9)			
Development agency	0 (0.0)	2 (7.7)	1 (3.8)	7 (26.9)	16 (61.5)			
Others	0 (0.0)	0 (0.0)	1 (25.0)	2 (50.0)	1 (25.0)			
Years of experience						22.256	0.035	0.136
< 5	2 (1.1)	2 (1.1)	14 (7.6)	53 (28.6)	114 (61.6)			
5–10	0 (0)	0 (0)	1 (1.4)	17 (23.6)	54 (75.0)			
11–15	1 (2.9)	0 (0)	0 (0)	14 (41.2)	19 (55.9)			
> 15	0 (0)	0 (0)	1 (0.9)	32 (28.6)	79 (70.5)			

Also, *χ* ^2^ analysis was undertaken to ascertain the relationship between sociodemographic characteristics and the view that the introduction of relevant tax waivers for the purchase of raw materials is a good strategy to improve local manufacturing. Compared to their contemporaries in the non‐pharmaceutical manufacturing group, more participants in pharmaceutical manufacturing group agreed with tax waivers with respect to the purchase of raw materials, but the difference was not statistically significant (*p* = 0.067, *V* = 0.148). However, a statistically significant but minimal relationship emerged for participants' highest educational qualification (*p* = 0.026, *V* = 0.139), age (*p* = 0.011, *V* = 0.141), and years of practice (*p* < 0.001, *V* = 0.178). The proportion of participants with more than 5 years of experience who agreed with the statement on tax waivers was higher than those with less years of employment history. Further details are presented in Table 3.

**Table 3:** Cross‐tabulation of demography with the statement “Introduction of relevant tax waivers for the purchase of raw materials is a good strategy to improve local manufacturing.”

Demography	Strongly Disagree (%)	Disagree (%)	Neutral (%)	Agree (%)	Strongly Agree (%)	*χ* ^2^	*p*	Cramer's *V*
Gender						0.523	0.971	0.036
Male	3 (1.2)	5 (2.0)	19 (7.5)	93 (36.5)	135 (52.9)			
Female	2 (1.4)	3 (2.1)	13 (8.9)	49 (33.6)	79 (54.1)			
Highest educational qualification						23.255	0.026	0.139
Diploma	1 (12.5)	1 (12.5)	0 (0)	3 (37.5)	3 (37.5)			
First degree	4 (1.5)	7 (2.6)	24 (8.9)	93 (34.6)	141 (52.4)			
Master's degree	0 (0)	0 (0)	3 (3.4)	33 (37.5)	52 (59.1)			
Doctorate	0 (0)	0 (0)	5 (14.3)	12 (34.3)	18 (51.4)			
Age (years)						31.781	0.011	0.141
18–30	4 (2.2)	5 (2.8)	21 (11.6)	68 (37.6)	83 (45.9)			
31–40	0 (0)	0 (0)	4 (4.7)	38 (44.7)	43 (50.6)			
41–50	1 (1.9)	0 (0)	1 (1.9)	15 (27.8)	37 (68.5)			
51–60	0 (0)	3 (4.6)	6 (9.2)	19 (29.2)	37 (56.9)			
Above 60	0 (0)	0 (0)	0 (0)	2 (12.5)	14 (87.5)			
Area of employment						8.779	0.067	0.148
Pharmaceutical manufacturing	0 (0.0)	3 (2.0)	6 (4.0)	57 (37.7)	85 (56.3)			
Nonpharmaceutical manufacturing	5 (2.0)	5 (2.0)	26 (10.5)	85 (34.3)	127 (51.2)			
Sector of practice						17.393	0.135	0.120
Government sector	1 (0.6)	6 (3.8)	18 (10.1)	62 (39.2)	73 (46.2)			
Private sector	4 (1.9)	2 (0.9)	11 (5.2)	67 (31.6)	128 (60.4)			
Development agency	0 (0)	0 (0)	3 (11.5)	12 (46.2)	11 (42.3)			
Others	0 (0)	0 (0)	1 (25.0)	1 (25.0)	2 (50.0)			
Years of experience						38.159	< 0.001	0.178
< 5	4 (2.2)	5 (2.7)	22 (12.0)	73 (39.9)	79 (43.2)			
5–10	0 (0)	0 (0)	3 (4.2)	34 (47.2)	35 (48.5)			
11–15	1 (2.9)	0 (0)	0 (0)	13 (38.2)	20 (58.8)			
> 15	0 (0)	3 (2.7)	7 (6.3)	22 (19.6)	80 (71.4)			

The cross‐tabulation further revealed that participants above 30 years were of the view that investment in research and development is important to improve the quality of medicines compared to those between 18 and 30 years (*p* = 0.021, *V* = 0.135). Similarly, respondents with over 5 years of experience shared the same view compared to participants with a shorter duration of experience, and this was significant (*p* = 0.009, *V* = 0.148). Further details are presented in Table 4.

**Table 4:** Cross‐tabulation of demography with the statement “Investment in research and development is important to improve the quality of drugs.”

Demography	Strongly Disagree (%)	Disagree (%)	Neutral (%)	Agree (%)	Strongly Agree (%)	*χ* ^2^	*p*	Cramer's *V*
Gender						4.676	0.322	0.108
Male	2 (0.8)	2 (0.8)	4 (1.6)	74 (28.9)	174 (68.0)			
Female	0 (0)	0 (0)	6 (4.1)	42 (28.4)	100 (67.6)			
Highest educational qualification						8.538	0.742	0.084
Diploma	0 (0)	0 (0)	0 (0)	1 (12.5)	7 (87.5)			
First degree	2 (0.7)	2 (0.7)	10 (3.7)	79 (29.0)	179 (65.8)			
Master's degree	0 (0)	0 (0)	0 (0)	26 (29.5)	62 (70.5)			
Doctorate	0 (0)	0 (0)	0 (0)	9 (25.7)	26 (74.3)			
Age (years)						29.530	0.021	0.135
18–30	2 (1.1)	2 (1.1)	10 (5.5)	51 (28.4)	117 (63.9)			
31–40	0 (0)	0 (0)	0 (0)	33 (38.8)	52 (61.2)			
41–50	0 (0)	0 (0)	0 (0)	16 (29.1)	39 (70.9)			
51–60	0 (0)	0 (0)	0 (0)	15 (23.1)	50 (76.9)			
Above 60	0 (0)	0 (0)	0 (0)	0 (0)	16 (100)			
Area of employment						6.003	0.199	0.122
Pharmaceutical manufacturing	0 (0)	0 (0)	3 (2.0)	37 (24.2)	113 (73.9)			
Nonpharmaceutical manufacturing	2 (0.8)	2 (0.8)	7 (2.8)	79 (31.7)	159 (63.9)			
Sector of practice						37.657	< 0.001	0.176
Government sector	0 (0)	0 (0)	5 (3.1)	48 (30.0)	107 (66.9)			
Private sector	2 (0.9)	0 (0)	4 (1.9)	65 (30.5)	142 (66.7)			
Development agency	0 (0)	2 (7.7)	0 (0)	2 (7.7)	22 (84.6)			
Others	0 (0)	0 (0)	0 (0)	1 (25.0)	3 (75.0)			
Years of experience						26.680	0.009	0.148
< 5	2 (1.1)	2 (1.1)	10 (5.4)	48 (25.9)	123 (66.5)			
5–10	0 (0)	0 (0)	0 (0)	24 (33.3)	48 (66.7)			
11–15	0 (0)	0 (0)	0 (0)	17 (50.0)	17 (50.0)			
> 15	0 (0)	0 (0)	0 (0)	27 (23.9)	86 (76.1)			

The analysis presented in Table 5 provides details of the association between the respondents' sociodemographics and perceptions regarding capacity‐building programs. The stakeholders' views of the statement that regular capacity building for manufacturers is critical to assure medicines security were significantly associated with age (*p* = 0.009, *V* = 0.143), although the effect size suggested a modest association between the variables. The findings revealed that a few of the younger participants, specifically those aged 18 to 40 years, disagreed with the statement. None of the older participants were in disagreement.

**Table 5:** Cross‐tabulation of demography with the statement “Regular capacity building for manufacturers is critical to assure medicines security.”

Demography	Strongly Disagree (%)	Disagree (%)	Neutral (%)	Agree (%)	Strongly Agree (%)	*χ* ^2^	*p*	Cramer's *V*
Gender						4.181	0.382	0.102
Male	3 (1.2)	0 (0)	16 (6.3)	97 (37.9)	140 (54.7)			
Female	0 (0)	1 (0.7)	9 (6.3)	60 (42.3)	72 (50.7)			
Highest educational qualification						20.750	0.054	0.132
Diploma	1 (12.5)	0 (0)	0 (0)	4 (50.0)	3 (37.5)			
First degree	2 (0.7)	1 (0.4)	17 (6.3)	98 (36.6)	150 (56.0)			
Master's degree	0 (0)	0 (0)	6 (7.0)	41 (47.7)	39 (45.3)			
Doctorate	0 (0)	0 (0)	2 (5.7)	13 (37.1)	20 (57.1)			
Age (years)						32.376	0.009	0.143
18–30	2 (1.1)	1 (0.6)	14 (7.8)	64 (35.8)	98 (54.7)			
31–40	1 (1.2)	0 (0)	6 (7.1)	41 (48.2)	37 (43.5)			
41–50	0 (0)	0 (0)	2 (3.8)	32 (60.4)	19 (35.8)			
51–60	0 (0)	0 (0)	2 (3.1)	20 (30.8)	43 (66.2)			
Above 60	0 (0)	0 (0)	1 (6.3)	0 (0)	15 (93.8)			
Area of employment						4.813	0.307	0.110
Pharmaceutical manufacturing	0 (0)	0 (0)	6 (4.1)	63 (42.9)	78 (53.1)			
Nonpharmaceutical manufacturing	3 (1.2)	1 (0.4)	19 (7.6)	94 (37.8)	132 (53.0)			
Sector of practice						3.757	0.987	0.056
Government sector	1 (0.6)	1 (0.6)	11 (7.0)	59 (37.3)	86 (54.4)			
Private sector	2 (1.0)	0 (0)	12 (5.7)	87 (41.6)	108 (51.7)			
Development agency	0 (0)	0 (0)	1 (3.8)	9 (34.6)	16 (61.5)			
Others	0 (0)	0 (0)	0 (0)	2 (50.0)	2 (50.0)			
Years of experience						15.619	0.209	0.114
< 5	2 (1.1)	1 (0.6)	15 (8.3)	68 (37.6)	95 (52.5)			
5–10	0 (0)	0 (0)	6 (8.3)	30 (41.7)	36 (50.0)			
11–15	0 (0)	0 (0)	0 (0)	21 (61.8)	13 (38.2)			
> 15	1 (0.9)	0 (0)	4 (3.6)	38 (34.2)	68 (61.3)			

## 4 Discussion

This study revealed novel insights into stakeholders' views on quality improvement and capacity‐building strategies for expediting medicines security in Nigeria. Given the cross‐sectional and perception‐based design, these findings reflect stakeholders' informed perspectives on priority areas within the Nigerian pharmaceutical system. The findings of this study show that regulatory agencies need to implement stringent measures to enforce compliance, particularly with GMP. This is strongly supported by the results, as the majority of the participants agreed that enhancing regulatory compliance was necessary, whilst almost all the respondents emphasised the need for stringent enforcement of GMP to ensure consistent product quality. This also aligns with contemporary literature emphasising the critical role of robust regulation on the quality of pharmaceutical products [14, 19]. Regulatory compliance plays a pivotal role in shaping the operational landscape of health systems globally and consequently impacts adherence to GMP, which is important for safeguarding patient health and maintaining public trust in healthcare products [24, 25]. Therefore, enhancing the implementation protocol for quality assurance would ensure the safety and efficacy of pharmaceuticals as well as reduce the economic liability associated with product recalls and litigation stemming from noncompliance. What this means is that the relevant National Regulatory Agencies need to increase monitoring and evaluation efforts, as this is necessary for the adoption of GMP, as well as for compliance with international standards. A major approach will be the adoption of adequate technologies to ease operations, enable data sharing, and thus enhance transparency and consistency with enforcement protocols [24]. Currently, Nigeria's progress toward the highest levels of WHO pre‐qualification is constrained by high capital costs required for GMP‐compliant upgrades, limited technical expertise on relevant requirements, and infrastructure gaps [26, 27]. Over‐reliance on imported active pharmaceutical ingredients and excipients further exposes local manufacturers to supply chain risks. Although Nigeria's attainment of WHO Maturity Level 3 represents a major regulatory achievement [28], sustaining the level of coordination needed for WHO pre‐qualification remains challenging. Furthermore, the strong agreement of better‐educated participants with improving enforcement of GMP, emphasises the need for the development of more capacity‐building activities in this area.

The majority of the participants in this study agreed that the implementation of tax waivers for the purchase of equipment and raw materials, as well as tax holidays by the government, was necessary to improve local pharmaceutical manufacturing capacity in Nigeria. The overwhelming support of the stakeholders in this regard is in accordance with findings in other countries where tax incentives have been instrumental in upscaling local manufacturing capacity [29]. For instance, in India, tax holidays and reduced tariffs on raw materials profoundly improved the competitiveness of local pharmaceutical firms, leading to improved quality and a reduction in the reliance on imports [30, 31]. Additionally, those in the pharmaceutical manufacturing sector were more inclined to agree to statements on tax waivers compared to those in other areas of practice. This may be attributed to manufacturers being more directly affected in these areas. It further highlights where targeted interventions focused on stimulating manufacturers' local production capacity should be directed for improved access to medicines by the public.

Over three‐quarters of the participants indicated that prioritising access to foreign exchange for local manufacturers was critical to support local manufacturing of medicines. The participants' views can be attributed to the prevailing foreign exchange challenges and the recognition of the difficulties faced by local manufacturers in sourcing critical pharmaceutical inputs and machinery, which are predominantly reliant on foreign currencies [32]. Beyond quality assurance, the challenges of accessing foreign exchange can further lead to increased exchange rates, thereby causing inflation in the prices of medicines [33, 34]. Therefore, there is a need for the government and relevant stakeholders to develop and streamline policies in this area so as to reduce unnecessary burden on local manufacturers.

The majority of the participants believed that the initiation of policies to mandate the government and its agencies to prioritise local pharmaceutical products can strengthen the sector. This further buttressed earlier findings that suggested that the government needs to be proactive in developing relevant policies for the local pharmaceutical industry [1]. Therefore, it will be critical for the government to ensure adequate implementation of its policies on prioritisation of local content by relevant ministries, departments, and agencies [35]. Moreover, there is also a need to promote locally made products to encourage public consumption [36]. Interventions targeted at promoting locally made medicines for the manufacturing sector can stimulate economic growth whilst contributing to broader public health outcomes of assuring access to affordable and high‐quality products in line with the medicines security concept [4]. Achieving this will depend on sustained adherence by manufacturers to international quality standards, as perceptions of inferior quality remain a major barrier to the acceptance of locally manufactured medicines [36]. Additionally, regulatory reforms such as streamlined approvals and harmonisation can support medicines security by minimising supply disruptions and improving continuity and equitable access to quality‐assured locally manufactured medicines [37].

Emergent findings also revealed that almost all the participants acknowledged the contextual need for public and private sector partnerships, with the respondents emphasising collaboration between government, industry, and other stakeholders to improve the quality of pharmaceutical products. Such partnerships can expedite resource distribution, as well as knowledge sharing between the government and the private sector, thereby significantly improving drug discovery and development activities [38].

In Nigeria, whilst there are existing alliances between government and private entities in the pharmaceutical manufacturing value chain, these collaborations are yet to produce optimal results for the populace [39]. This may be as a result of fragmented governance structures, weak coordination between government, academia, and industry, and the absence of enforceable accountability mechanisms. Additionally, misalignment between research priorities, industrial capacity, and public health needs, alongside regulatory and financing bottlenecks, has constrained the translation of collaborative efforts into sustained research and development outputs [40, 41]. Therefore, robust and innovative measures can be adopted to ensure that partnerships with the private sector translate into effective interventions that can drive contextual development. However, translating collaborative intent into tangible outcomes requires more rigorous policy design. One approach is the use of conditional incentives. Under this arrangement, fiscal benefits such as tax credits, duty waivers, as well as research and development grants are tied to clearly defined performance targets. These targets may include investment in locally relevant innovation, technology transfer, or measurable progress toward the WHO pre‐qualification. Aligning incentives with clearly defined performance targets may improve accountability and ensure that public support directly contributes to innovation, quality improvement, and access. In addition to collaborative partnerships, the majority of the participants highlighted that increased government investment in research and development was critical in driving innovation. This suggests the need for prioritisation of funding for research and development activities to bolster innovation and provide sustainable solutions that align with local challenges. The lack of innovative research in the country's pharmaceutical manufacturing sector is largely due to inadequacies in funding and contextual investments [12]. Investment in pharmaceutical research and development, therefore, strengthens medicines security by expanding the pipeline of safe, effective, and context‐appropriate products, including for priority and neglected diseases. Evidence from the WHO [42] indicates that sustained, needs‐driven research and development improves the availability, affordability, and timely access to essential medicines, thereby reducing dependence on limited or outdated therapeutic options.

Furthermore, a strong majority of the participants indicated that there was a need for relevant capacity‐building programs for manufacturers to support medicines security. Also, the participants emphasised that regular training on WHO pre‐qualification is requisite to ensure that medicines meet international quality standards. This is similar to previous findings [20, 43]. Additionally, more of the older participants indicated that regular capacity building for manufacturers was critical to assure medicines security compared to younger participants. This can be linked to older persons having more robust experience and recognising the value of continuous human resource development for maintaining a competitive and adequate workforce in the healthcare system.

The priority areas identified in this study align with findings from research and policy analyses in other low‐ and middle‐income countries, where strengthening regulatory systems and quality assurance is critical for ensuring access to safe and effective medicines. For instance, in Ethiopia, regulatory capacity constraints and a limited skilled workforce have hindered local pharmaceutical production scale‐up [44]. Similarly, Bangladesh and Kenya have demonstrated that structured investment in GMP compliance, workforce training, and regulatory enforcement significantly improves the quality of medicines as well as the continuity of supply [42]. In Pakistan, government‐led initiatives to improve access to active pharmaceutical ingredients and strengthen local manufacturing policies show that aligning fiscal incentives with quality standards can enhance both availability and affordability of essential medicines [45]. These suggest that the challenges and priorities observed in Nigeria are consistent with broader low‐ and middle‐income countries' experience, emphasising the importance of regulation, capacity building, as well as research and development for medicines security. Overall, the findings indicate that stakeholders perceive medicines security as an outcome of interrelated regulatory, economic, research, collaborative, and capacity‐building efforts rather than isolated interventions.

This study is not without limitations. For instance, despite the pioneering role of the research, the data collection approach which relies on self‐reported perceptions may introduce social desirability bias which may in turn affect the generalisability of the findings.

## 5 Conclusion

This study provides stakeholders' perspectives on the role of quality improvement and capacity‐building initiatives in expediting medicines security in Nigeria. The findings highlight key areas where strategic interventions should be directed. These include: regulatory strengthening, quality assurance systems, and capacity building. Others are investment in research and development, procurement policies, and structured public–private collaborations.

From the findings, improvement measures such as stricter regulation and enforcement of GMP, as well as regular training for the WHO pre‐qualification, are pivotal to assure quality of medicines produced locally. It also emerged that implementation of economic policies such as reduced taxes on pharmaceutical raw materials and production equipment, in addition to the prioritisation of local content, are crucial to upscaling domestic manufacturing capacity.

The study further revealed that government investment, particularly in research and development, should be optimally increased to drive innovation in the pharmaceutical manufacturing sector. Within this context, quality assurance–related priorities such as regulatory enforcement, GMP compliance, and skills development were perceived as foundational to ensuring the quality and safety of medicines. At the same time, production‐capacity–related priorities, including local manufacturing scale‐up, financing mechanisms, and market‐shaping policies, were viewed as essential for maintaining supply continuity and equitable access. Together, these interconnected but distinct domains reflect stakeholders' perceptions of practical pathways for assuring medicines security in Nigeria.

Accordingly, these findings can support institutional reforms that guide policymakers and regulators in translating stakeholder priorities into practice. These can be achieved by strengthening risk‐based regulatory enforcement, aligning fiscal incentives with quality compliance, and embedding capacity‐building initiatives within existing health and industrial policy frameworks. Future research could employ longitudinal and intervention‐based designs to evaluate the effects of regulatory reforms, economic incentives, and capacity‐building initiatives on medicine quality, availability, and local manufacturing performance over time. Intervention studies assessing the impact of structured training and the WHO pre‐qualification support on quality management outcomes are particularly warranted. In addition, mixed‐methods approaches could provide deeper insights into contextual, institutional, and financial barriers that influence implementation and sustainability.

## Author Contributions

**Obi Peter Adigwe:** conceptualisation, investigation, methodology, validation, formal analysis, data curation, supervision, writing – review and editing. **Godspower Onavbavba:** methodology, investigation, validation, formal analysis, project administration, data curation, writing – original draft. **Toluwalope Folashade Alabi:** methodology, validation, investigation, writing – original draft, formal analysis, data curation. **Nwakaego Rosemary Uzuh:** investigation, methodology, formal analysis, data curation, writing – original draft. All authors have read and approved the final version of the manuscript.

## Funding

The authors have nothing to report.

## Ethics Statement

Ethical approval was sought and obtained from the National Institute for Pharmaceutical Research and Development Health Research Ethics Committee (Approval Number: HREC 09/11/2024‐33).

## Consent

Each participant provided a written informed consent that validated their voluntary participation.

## Disclosure

Obi Peter Adigwe affirms that this manuscript is an honest, accurate, and transparent account of the study being reported, that no important aspects of the study have been omitted, and that any discrepancies from the study as planned (and if relevant, registered) have been explained.

## Conflicts of Interest

The authors declare no conflicts of interest.

## Supporting information


Supporting File

## Data Availability

Data will be made available on request. The authors confirm that the data supporting the findings of this study are available within the article. Obi Peter Adigwe has full access to all of the data in this study and takes complete responsibility for the integrity of the data and the accuracy of the data analysis.
