# Supplementary material for: The Role of Research, Quality Improvement, and Training in Achieving Medicines Security in Nigeria: A Cross‐Sectional Exploration of Stakeholders' Perspectives
Source: Health Sci Rep. 2026 Aug 4;9(8):e72624. doi: 10.1002/hsr2.72624 (PMC13434838; doi:10.1002/hsr2.72624)
Supplement: Supplementary file 1 [file HSR2-9-e72624-s001.docx]

**The Role of Research, Quality Improvement and Training in Achieving Medicines’ Security: An Empirical Exploration of Stakeholders’ Knowledge and Experience**

**Introduction**

To achieve growth in national capacity for sustainable manufacturing of medicines, quality improvement and capacity building in the Nigerian pharmaceutical industry are critical. This study aims to assess your views and perception on the role of quality improvement and capacity building in enhancing pharmaceutical industries to meet the medicines’ need of the country. Please fill out the questionnaire by ticking (√) the most appropriate option (s). Your responses will be anonymised and treated confidentially.

**Section A: Demographic Data**

**1. Gender**

| Male | Female |
| --- | --- |

**2. Age (years)**

| 18 -30 | 31-40 | 41-50 | 51-60 | Above 60 |
| --- | --- | --- | --- | --- |

**3. Highest Educational Qualification**

| Diploma | First degree | Master’s degree | Doctorate |
| --- | --- | --- | --- |

**4. Years of Experience in Your Current Work**

| ˂5years | 5-10years | 11-15years | Above 15years |
| --- | --- | --- | --- |

**5. Sector of practice**

| Government Sector | Private Sector | Development Agency | Others, please specify ………………………………. |
| --- | --- | --- | --- |

**6. Area of Employment**

| Pharmaceutical Manufacturing | Non-Pharmaceutical Manufacturing |
| --- | --- |

For the following statements in Sections B and C , please give your rating: SA (Strongly Agree), A (agree), N (Neutral), D (disagree), and SD (strongly disagree).

**Section B: Strategies for Improving the Quality of Pharmaceutical Manufacturing in Nigeria**

| **SN** | **Statement** | **SD** | **D** | **N** | **A** | **SA** |
| --- | --- | --- | --- | --- | --- | --- |
| 1 | Enforcement of Good Manufacturing Practices by the pharmaceutical regulatory authorities is critical to ensure consistent drug product quality. |  |  |  |  |  |
| 2 | Investment in Research & Development is important to improve the quality of drugs produced. |  |  |  |  |  |
| 3 | Regulatory oversight needs to be enhanced to ensure compliance with quality standards. |  |  |  |  |  |
| 4 | Collaboration between the government, pharmaceutical industries, and other stakeholders is critical to improving quality of drug products. |  |  |  |  |  |
| 5 | Introduction of relevant tax waivers for the purchase of equipment is a good strategy to improve local manufacturing. |  |  |  |  |  |
| 6 | Prioritising access to foreign exchange for local manufacturers is critical to support local production of medicines. |  |  |  |  |  |
| 7 | Providing tax holidays is an important strategy for industries’ improvement initiatives. |  |  |  |  |  |
| 8 | Prioritisation of the pharmaceutical sector and relevant agencies within the industry by government is critical to achieve Medicines’ Security. |  |  |  |  |  |
| 9 | The initiation of purpose built pharma parks can improve access to medicines. |  |  |  |  |  |
| 10 | Initiation of policy to mandate government and its agencies to prioritise local pharmaceutical products can strengthen the sector. |  |  |  |  |  |
| 11 | It is important for development partners to prioritise procurement of local pharmaceutical products over foreign alternatives. |  |  |  |  |  |
| 12 | Introduction of relevant tax waivers for the purchase of raw materials is a good strategy to improve local manufacturing. |  |  |  |  |  |

13. What other views do you have about strategies for improving quality of pharmaceutical manufacturing in Nigeria?............................................................. …………………………………………………..………………………

**Section C: Perception on how Quality Improvement Programmes can help Assure Medicines’ Security**

| **SN** | **Statement** | **SD** | **D** | **N** | **A** | **SA** |
| --- | --- | --- | --- | --- | --- | --- |
| 1 | Regular training on the WHO pre-qualification is required to ensure that medicines meet international quality standards. |  |  |  |  |  |
| 2 | A WHO pre-qualification is necessary to guarantee access to high-quality essential medicines. |  |  |  |  |  |
| 3 | The proliferation of low-quality pharmaceuticals in Africa can be controlled by regular training on areas where gaps exist. |  |  |  |  |  |
| 4 | Prioritisation of manpower development can improve the standard of healthcare in Africa. |  |  |  |  |  |
| 5 | Public confidence in locally produced medicines can be increased by attaining WHO pre-qualification. |  |  |  |  |  |
| 6 | The globalization of African pharma markets will be facilitated by WHO pre-qualification attainment. |  |  |  |  |  |
| 7 | Regular capacity building for manufacturers is critical to assure Medicines’ Security |  |  |  |  |  |

8. What other views do you have regarding how quality improvement programmes can help assure Medicines’ Security in Nigeria?

....................................................................................................................................

**Thank you for taking the time to complete this questionnaire**
